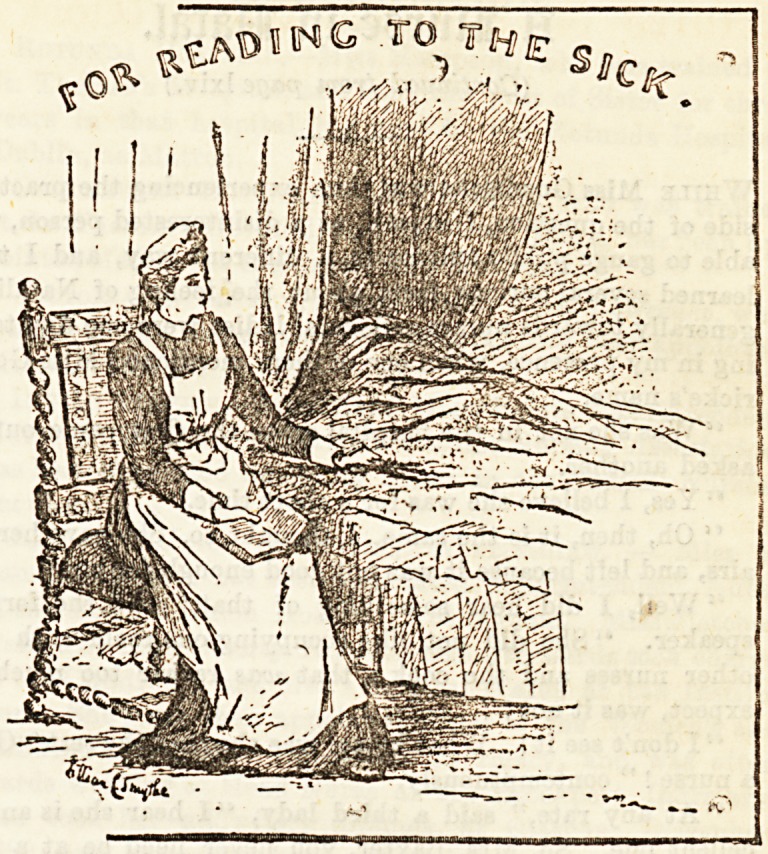# The Hospital Nursing Supplement

**Published:** 1891-12-26

**Authors:** 


### "The Hospital" Nursing Mirror.

Being the Extra Nursing Supplement of "The Hospital" Newspaper.

Contributions for this Supplement should be addressed to the Editor, The Hospital, 140, Strand, London, W.C., and should have the word "Nursing" plainly written in left-hand top corner of the envelope.

### En Passant.

SHORT ITEMS.—Several nurses lately attended Mrs. Jopling Rowe's studio, when a lecture on anatomy was given, W. Sandow as a model.—Mr. Malleson has been addressing a drawing-room meeting at Bath, in favour of the district nurses; she wants them to affiliate with the Jubilee Institute and have the nurses living in a central home under a Lady Superintendent.—A nurse writes from Paris to say that work is very slack there, and she warns her fellow workers from being deluded into crossing the Channel unless a settled salary is promised them in writing.

QUIET COURAGE.—The latest heroine (says the *Women's Journal*, of Boston) is Sister Margaret Frances, of St. Victor's Convent, near Montreal. The convent contained a large number of deaf-mute children as pupils, but had no fire-escape, although the children were lodged on the sixth floor. At an hour in the morning when sleep is the soundest, Sister Margaret, in charge of the sixth floor dormitory, awoke to find the room on fire and the children flocking around her. The stairway was on fire, and the would-be rescuers who came from the neighbourhood had no ladders. A worse outlook for escape could scarcely be imagined; yet this one woman saved the children and herself too. She gathered them in the corner of the building farthest from the flames, and, making a rope of bed clothing, lowered her charges one by one to the ground. She kept at her work for half an hour, until the last child was safely landed; then slipped down the rope herself, with her hands and arms blistered by the heat and the hair singed from her head, and fainted when she reached the ground.

A CANADIAN SCHOOL.—They seem to be a frivilous race "across the pond" to judge by this account of what would, in England, be a solemn and a sad undertaking. "A chorus of college songs, a confused medley of 'He's a Jolly Good Fellow,' 'Litoria,' and the angelic 'Clementina,' greeted Mr. Walter S. Lee yesterday afternoon as he stepped to the table in the theatre of the Toronto General Hospital, and opened the proceedings of the commencement exercises of the class of 1891 Toronto General Hospital Training School for Nurses. The seats were well-filled with bright-faced nurses who had vivid recollections of the time when they had to undergo the ordeal, and nurses undergoing the training of the school in their neat uniforms and white muslin caps, with here and there a black band, showing that the wearer had risen to the dignity of a head nurse, lady friends of the graduates, and in the upper tier the medicos were gathered in full force to cheer the speakers and the graduates." This refers to the annual distribution of medals and diplomas to the nurses of the Toronto General Hospital. The proceedings were varied by songs and recitations and by an excellent speech from Dr. O'Reilly, to whom, and to Miss Snively, the Matron, the success of the training school is largely due. Dr. O'Reilly expressed a hope that some day a batch of Canadian-born and Canadian-trained nurses would receive the honour, as members of the American Pension Fund, of being presented to the Princess of Wales. They seem to appreciate the Pension Fund at Toronto, and to have taken up the subject with all the "go" which marks the younger races of the world. On the part of the Two Thousand Pension Fund nurses in England, we can offer our Canadian sisters a hearty welcome when they come to visit us. There are 131 holders of the diploma of the Toronto Training School for Nurses, presented after two years of service.

A HAPPY CHRISTMAS!—To all our readers far and near we send our best wishes for a joyful Christmastide. Though many dwell in the midst of sad scenes and see all the bitterness and misery of life, they see also all the love and self-sacrifice of which humanity is capable, and they can keep with joy the birthday festival of Christ, who was "made perfect through suffering." Some of our constant correspondents, both in the colonies and at home, have become very true friends to us and to The Hospital; it is with real gratitude and affection we now send them our best wishes, being denied the pleasure of grasping them each by the hand.

SCOTTISH QUEEN'S NURSES.—The third annual report of the Queen Victoria Jubilee Institute, Scottish Branch, tells of great progress during the past year. The demand for nurses has been so great that five new cubicles have been arranged, and the Home can now hold sixteen nurses. During the year twelve local associations had been formed in connection with the institute, and had engaged Queen's nurses. During the year 1,114 cases had been nursed, and 29,528 visits paid. The accounts showed that the receipts for the year amounted to £1,989 7s., and the expenditure to £1,502 18s. 6d., leaving a surplus for the year of £486 8s. 6d. Lectures have been arranged for the nurses. The report, which is signed by Princess Louise, concludes with a tribute of praise to Miss Peter for her devoted work.

AUSTRALIAN NOTES.—"Nothing was ever more delusive than the idea that the influenza would never be able to cross the equator. It has come in force, and in force it remains. Its action has been sporadic, and apparently most arbitrary, and wherever it has appeared it has played havoc. Doctors, judges, clergy, Ministers of the Crown, and all other estates and conditions of men have been laid low by it impartially. Several of the public departments reached everything short of paralysis for want of officers. Many of the country districts were worse off than Melbourne. The good people at N—— sent urgent telegrams imploring medical aid, but as every one was engaged a dozen deep only a nurse could be sent. 'I don't know very much,' was her remark on her return, 'but, you see, they didn't know anything at all.' After a long day's journey she arrived at the house at which she was to stay. Every one of the inmates was down with the influenza. She attended to her hosts (?) and was then driven from house to house in the neighbourhood, and in every house found patients. Amateur nursing is often funny—sometimes fatal. In one house our nurse found a magnificent young fellow in consumpton accompanied by hæmorrhage. His friends were keeping him seated bolt upright and giving him hot beef tea! The action taken by the Committee of the Austin Hospital for Incurables respecting occasional entertainments given by the Fernside Bicycle Club has had a damaging effect, and the patients are deprived of a great and most welcome pleasure. Our Hospital Sunday has not been so successful as usual, while the Melbourne Hospital itself is more in debt that ever. On November 6th, the degree of M.B., was conferred on Miss Clara Stone, and Miss Margaret Whyte, the first successful women students of Medicine in the University of Melbourne. On presentation to the Chancellor, they received quite an ovation. The former of these young ladies has a sister holding three diplomas, and now in extensive and successful practice in Melbourne."—*From Our Own Correspondent.*

### Lectures on Surgical Ward Work and Nursing.

By Alexander Miles, M.D. (Edin.), F.R.C.S.E.

### Lecture XL.—URETHRAL INSTRUMENTS.

Bougies.—These instruments are used for the dilatation of the urethral canal. They are made of various materials, some rigid, others soft and flexible, and of different shapes, but all agree in being solid, or at least in not permitting of the flow of urine through them. They derive their name from the fact that wax tapers or "bougies" were previously used in place of specially-made instruments. They are about twelve or thirteen inches long, and vary in diameter according to a set scale, each instrument having its size marked on it.

(1) The ordinary silver bougie resembles the silver catheter in every respect, save that it has no eye at the point, and is impermeable. It is of the same diameter throughout.

(2) Sir Joseph Lister's silver bougies are solid, and therefore of considerable weight, which greatly assists their passage along the canal, and so renders force on the part of the surgeon unnecessary. These instruments are graduated so that the diameter increases by three sizes from the point upwards. Hence they come to be referred to as "Lister's 2-5" or "10-13," and so on, which means that just beyond the point (which is made slightly bulbous for safety) the size is No. 10, and at the middle of the stem it has increased to No. 13. Of rigid instruments these are usually accepted as the best.

Various makes of supple instruments are in use, for example, the English gum elastic bougie, which is made of finely-woven silk, covered with several coats of brownish copal varnish. It is of the same diameter throughout, and is rendered quite supple by being warmed to the temperature of the body. It may be made to take any "set" desired by being left bent for a few weeks. (Fig. 1.)

(4) The French Gum Elastic Bougie differs somewhat in shape from the English. It is made so that the stem gradually tapers away to a point in its lower third. An improvement on this is to have the tip rounded and slightly bulbous, which prevents "hitching" on any obstruction in the canal. This is the "Bougie à Boule" of the French. These instruments are black. (Fig. 2.)

(5) The "Acorn" Bougie is mainly used as a diagnostic instrument. It consists of a long slender stem, fitted at the point with an acorn-shaped bulb. This is so formed that it passes pretty readily in through a stricture, but is distinctly caught on being withdrawn. In this way the exact position of the stricture may be determined, and then by using instruments of different sizes, an idea of the calibre of the tube at the strictured point may be obtained. (Fig. 3.)

(6) The "Filiform," or thread-like bougie, as it name implies, is of very fine calibre, and is chiefly employed in cases of exceeding tight stricture, to determine if the passage is at all permeable or not. If once passed it facilitates the passage of larger instruments. (Fig. 4.)

Other instruments, such as (7) the Ball-staff Bougie; (8), Bougie à Ventre; (9) Bougie à Ventre à boule, are less frequently used.

Catheters.—These instruments are hollow tubes made of different materials, and are used to empty the bladder. They are to be distinguished from bougies which are solid, and are for the dilatation of the urethra. Almost each variety of bougie which we have already described, has its counterpart in the shape of a catheter.

(1) The ordinary silver catheter (Fig. 5) consists of a hollow tube, with a small "eye" on its side, about a quarter of an inch from the point. In the older instruments the part of the catheter beyond the eye formed a cul-de-sac in which dirt, and therefore septic material, was apt to collect, and constituted a source of danger by introducing organisms into the urine, which is a highly putrescible fluid. In more recent days this disadvantage has been removed by making the point of the instrument solid. Near the other end of the tube there are two small silver rings, which are used when the instrument has to be tied into the bladder, for constant drainage. Most catheters are furnished with a wire stylet. The lower fourth of the instrument is curved so that the point comes to

Fig. 1. Fig. 2. Fig. 3. Fig. 4. Fig. 5. Fig. 6. Fig. 7. Fig. 8.

be almost at a right angle with the stem. The sizes, according to the English scale, are from ½ to 12.

The other catheters are all more or less flexible. (2) The rubber catheter is quite soft, and is employed when there might be danger of a more rigid instrument doing harm; for example, in old men who require to use the catheter habitually, and who have to pass it themselves.

(3) Gum-elastic Catheters (English and French) resemble bougies of corresponding makes. They are quite flexible when heated to the temperature of the body, and are supplied with a wire stylet to give them the necessary amount of rigidity, or the appropriate curve when being used.

(4) The Catheter à Coudée, or elbowed catheter, is also made of gum elastic. About half an inch from the beak it is bent at an angle of 45 deg. with the stem, and this bend is permanent. It is employed in cases of enlarged prostate. (Fig. 6.)

(5) The Catheter Bi-Coudée has a double bend near the beak.

(6) The Filiform catheter is used to empty the bladder in cases of very tight stricture.

(7) Female catheters (Fig. 7) are short, with a slight curve. They are seldom used, the ordinary gum elastic male catheter being much more serviceable.

(8) Various instruments have been employed for introducing caustics and other medicaments to the urethra, but they are seldom used nowadays.

(9) The Double-barrelled catheter (Fig. 8) is used to wash out the bladder, the lotion passing in along one channel, and out along the other. It may be used with a syringe or syphon.

(10) Other forms of catheter are used in certain operations, e.g., lithotrity, where the bladder has to be washed out to remove all fragments of the stone. Examples of these are Clover's and Bigelow's evacuating catheters, the chief characteristics of which are their large bone and the size of the eye, which is much larger than in ordinary instruments, to permit of the passage of stones.

The illustrations are used by permission of Messrs. Maw, Son, and Thompson.

### Christmas Competitions.

FIRST of all a thousand thanks to our many readers who have sent us parcels of clothing either for distribution or in competition. Owing to their kind co-operation we were able to give away to various hospitals 70 pairs of socks, 40 warm petticoats, 10 blouses, 5 shirts, 5 cross-overs, 3 shawls, 2 pinafores, 6 children's petticoats, 4 vests, 20 pairs of mittens, 10 cravats, 2 hug-me-tights, 8 pairs of stockings, 2 nightingales, 2 pairs of knitted gloves, 1 pair of hand-worked slippers, some underlinen, and last, but not least, seven dressing-gowns. It was delightful work opening the parcels, and the writer was helped by Miss Gordon, Matron of Charing Cross, and Miss Pritchard, this little Committee of three having undertaken the difficult task of judging the work sent in. The socks were first attacked and examined as to shape, to workmanship, and to general appearance; it was very hard to decide which pair was actually the best, but at last the Committee awarded prize No. 1 to NURSE A. FRITH, while they gave honourable mention to Nurse E. Dudley, Nurse Mary Trump, Nurse Corner, and Nurse Chandler. The second prize went to MISS MABLE WHITE, though Mable Gillian, a little girl of twelve, ran her very close. MISS HALE, of Rotherham, took the prize for a flannel shirt, and we regret more of our readers did not enter for this third competition; shirts are so very useful to give away, though difficult to make. The fourth competition was won by MISS MCEWEN, while a charmingly-made check blouse, from Miss Clarke, was greatly admired. The flannel petticoat competition was very difficult to judge; at least six of the petticoats sent came very near perfection, but in the end the prize was given to MISS G. KEMP, while Miss R. Parsons received high commendation. For the dressing-gown competition there were only five entries, and the prize was awarded to a grey check gown made by NURSE AYRTON, which was lined with flannelette, and of good shape. One nurse sent a very cleverly-cut gown for a helpless patient, and Miss Clarke sent one which was all woollen, even to the trimming. We shall be glad to hear from those prize winners who would prefer books to money; perhaps they would kindly state what books they want. The story of the distribution of the parcels will be told next week, for even while we write more parcels are coming in to swell the list of clothing we have to give away. Meanwhile, we are sure that all those who spent part of their holiday time in making these warm garments will have an extra joy on Christmas morning, when they think that some one sick in a strange ward has been cheered by their gift.

### A MERRY CHRISTMAS!

EVERY where around us we hear the usual phrase, "A Merry Christmas to you." To-day the air is full of the sound of sweet bells, which call us early to worship the Babe of Bethlehem; and the glad tidings are published far and wide, of "Peace on earth, goodwill towards men." We wish you a merry Christmas, say our friends, and perhaps we feel they are mocking us who cannot get out of bed and enjoy ourselves in any jolly way. Yes, a Merry Christmas, again they repeat, and just because you *are* lonely and sick and miserable we say it with the heartier good will. Of course by "merry" they do not mean rioting and drunkenness, loud laughter and foolish jesting which is not convenient, but that sober, cheerful mind which the wise man describes in the words, "A merry heart doth good like a medicine"—a medicine which both heals and strengthens itself and its neighbours. And at Christmastide how many kind souls there are who think of their suffering fellow creatures, and supply them with the means of having a few hours of social happiness and enjoyment. Even in the sick ward of a hospital many weary hearts are cheered and comforted by the feeling that they are not forgotten by their healthier and wealthier brethren, but share as far as their strength will admit in the general rejoicing. So, as iron sharpeneth iron, we will try and forget our troubles for the moment, and join in singing "Peace on earth, goodwill towards men."

But we will not forget the true rejoicing which ought to fill our hearts, especially this day when we are keeping the birthday of our blessed Lord. We turn to Him who laid aside all the glory of the King of Heaven, and condescended to be sheltered in a stable and cradled in a manger. Why did he come in this strange and poor disguise?

"He came the broken heart to find,  
The bleeding soul to cure,  
And, with the treasures of His grace,  
To bless the humble poor."

Angels proclaimed through the frosty, silent night, some 1,900 years ago, that God had come down on earth to dwell, and,

"Like circles widening round  
Upon a clear blue river,"

the wondrous message of "Love towards men of love" has reached through and round the world to us. Christ came in humility, and it is humble, loving souls with whom he loves to dwell. The single heart, the virgin mind, these are what we must bring to worship him, and having welcomed and adored Him, we shall grow contented and merry (in the right sense), and henceforth join in singing, "Glory to God in the highest; on earth, Peace, goodwill towards men.

### A Nurse in Natal.

(*Continued from page* lxiv.)

While Miss Goodricke was thus experiencing the practical side of the question, I myself, as a disinterested person, was able to gauge public opinion in a different way, and I thus learned several curious facts about the feeling of Natalians generally towards nurses. Several ladies were one day talking in my presence, when one of them mentioned Miss Goodricke's name.

"Was she not in the hospital when she first came out?" asked another.

"Yes, I believe she was for a short time."

"Oh, then, it is the same. I thought so. She gave herself airs, and left because it was not good enough for her."

"Well, I did hear something of that," said the former speaker. "She did not like occupying one room with two other nurses and the cook; that *was* rather too much to expect, was it not?"

"I don't see it! What better was she than the rest? Only a nurse!" contemptuously.

"At any rate," said a third lady, "I hear she is an excellent one. So, Mrs. Baxter, you never need be at a loss for a good nurse in the future."

"I!" exclaimed Mrs. Baxter. "I'll have none of your trained nurses. There'll never be a nurse in my house as long as I have a word to say in the matter!"

"I hear," said another, "that Miss Goodricke does not care in the least for dances or tennis, or anything of that sort. Seems queer, does it not, to give up everything for the sake of nursing sick people. I think there is something very odd about it." This was said in that slightly mysterious tone which often does so much mischief.

"At any rate," broke in a young girl of the company, "she must be a great donkey to do it!"

Before quitting this part of my subject I may perhaps give a brief reference to two cases which Miss Goodricke undertook in Myburg, as they illustrate the different classes of whom mention has been made. One of these patients was the wife of a man in easy, though not affluent, circumstances, living a little out of town. She had always been in the habit (from choice, not necessity) of doing her own household work, and she seemed quite surprised when Miss Goodricke asked what servant she had.

"Oh, I don't keep one," she said. "I do everything myself."

"But," said Miss Goodricke, "I do not see how I am to give the proper attention to you if I have everything not only to look after, but to do, in the house. What about your husband's meals? Can he take them out for a time?"

"Certainly not. I would not hear of such a thing. You will have to manage that; and he is obliged to have them very punctually, too."

"And your children, Mrs. Smith—(there were two little mites, besides a baby)—what about them at night? Can they sleep alone?"

"Of course not, they are much too young. I don't see what you can do but make them up a bed in this room, and look after them yourself."

"And the house-cleaning and fires, I do not know how I shall manage," said the bewildered nurse. "I have never had to chop wood or carry water in my life."

"Well, perhaps we may get in a Kaffir to do that now and then," said Mrs. Smith, in a grudging tone; but, as a matter of fact, no Kaffir was procured.

It must be borne in mind that housework in a semi-tropical climate, and with the scant conveniences which Natal still possesses, is a very different thing from what it is in England. In this case fresh water had to be brought from a pump about thirty yards from the house, and all refuse to be carried a distance of a quarter of a mile away. While she was scouring the "pots" in the back yard, she was distracted by the knowledge that her patient needed her, that the baby was screaming at concert pitch, and the little three-year old twins were rolling in the gutter that ran before the house. Not to mention the harassing thought that the husband would be home punctually to his half-past twelve dinner, and would be very short in his manner if it were not ready to the minute. My English readers will not be surprised to hear that after leaving this patient convalescent Miss Goodricke was laid up for a fortnight in consequence of the overstrain and fatigue. Of course, this was rather an extreme case, and in glowing contrast to it was the next, which came in her way after six weeks' inactivity. This family lived in one of the suburban districts of Myburg; they had ample means, and every comfort and refinement which money and the country could compass. It was not, however, these accessories only which made her residence there a pleasant and grateful memory.

It was chiefly that the whole family, realising her importance to one of its members, treated her with unvarying kindness and attention. She was not allowed to do anything outside of the sick room, and, beside this, the mistress of the house took care that she should have plenty of rest and fresh air by frequently taking her place at the invalid's bedside. "You seem to have a very different opinion of trained nurses from most people in Natal," Mary could not refrain from once saying.

"Perhaps I have," said Mrs. S., smiling. "But to my mind the principle involved in engaging a nurse is the same as that in engaging a governess—I want the best I can get. When I needed a governess for my children I did not seek for an uneducated young girl, who was to make up for her educational deficiencies by being seamstress and general help in the house, as so many people do. I was not satisfied until I had obtained a thoroughly educated and refined lady as my children's instructress and model. And so with a nurse. If my husband or one of my children is ill I do not want to risk (perhaps) their life by putting them in the hands of a woman who, probably, with the best intentions in the world, knows nothing whatever about her business. That is why I begged Dr. Henders to secure your services for us, if possible, when Maggie was taken ill."

(*To be continued.*)

### A Christmas Thought.

"What a thought for these last days—when we know ourselves to be going home to God, God Whom at bottom, after all, we have loved and shall love for ever—that we *might* have better served Him here, *might* have blessed His creatures, *might* have done His will on earth as it is done in heaven, but we have let many glorious chances slip by us for ever. By giving your hearts to your cause of charity you will learn thereby precious secrets. . . . . You will learn how noble are the friendships which spring out of common work in unselfish and holy tasks; friendships to which the comradeships of social pleasures are as dross to gold. And you will learn what it is to feel, when the evening of life closes in—whether you have or have not been allowed to 'see of the travail of your soul'—that you have done what in you lay to bring relief to the suffering, guidance to the erring, restoration to the fallen, and justice to the wrong and miserable. In other words, you will feel that you have not only *prayed* to God, but *lived* the prayer, 'Thy kingdom come.'"—From *Women's Duty to Women*, by F. P. Cobbe.

### Everybody's Opinion.

### NURSING AT THE LONDON HOSPITAL.

DORA SCRIMGEOUR writes: Will you allow me to correct an error in your issue of the 5th inst., in which you state that I was for three months a probationer in the London Hospital, and to make some reply to your observations on my resolution in favour of a three weeks' holiday for nurses? I never worked in the London Hospital, although several of my friends and relations have been probationers there. But it does not require even my small acquaintance with hospital life to enable me to form an opinion on this subject. I am guided by the hospital authorities themselves as to the desirability of extending the holidays. As to the practical possibility of so doing, I am furnished with data by the Matron. The calculation seems to me very simple. Given 168 nurses and probationers to be allowed one extra week's holiday in the year, four additional nurses would more than supply the deficiency. And the cost to the hospital would be simply their salaries. It is obvious that no additional board or lodging need be provided. It would have have been more to the purpose if, instead of meeting this arithmetic with "smiles and silence," the Committee had met it with a clear refutation. Are they not leaving the uninitiated to infer that "Silence gives consent" in this case and that my line of reasoning was incontrovertible? Surely the falling-off in the subscriptions to which you allude is a proof that the charges against the London Hospital never have been refuted. Let the Committee show that the complaints were without foundations, or if this is not possible, let them give us their programme of reform, and they may then have reason to expect a return of that public confidence the loss of which is so much to be regretted.

[We regret that through an error we credited Miss Scrimgeour with even a short practical acquaintance with the subject in hand.—ED.]

### GLASGOW ROYAL INFIRMARY.

"ONE OF THE NURSES" writes: The nurses of the above institution cannot speak too highly of the marked improvement that has taken place in their condition since Mrs. Strong was elected Matron. If they have been rather tardy in acknowledging this publicly it has by no means been due to a want of appreciation on their part. The air of contentment that reigns among the nurses now is one of the best proofs of how satisfactory the present state of affairs is, and also of how much needed the reform was.

[Most gladly do we welcome to the ranks of the contented our hard-working Scotch sisters. May they be absolutely loyal to the Matron who is doing her best for them.—ED.]

### LOCAL HIGH TEMPERATURE.

"LADY-IN-CHARGE" writes: I am sorry I cannot send you further information at present, but the case is taken up by the Exeter Medico Chirurgical Society, and the woman is in the Devon and Exeter Hospital very ill, but not likely to yield a satisfactory solution to the problem of her most extraordinary temperature yet. I saw her yesterday, and it had been taken 105 deg., and then found to be a very much lower one. I will if possible let you know any further particulars or send you any papers that may be published. My facts were accepted as such. Dr. Day and Dr. Gordon have the case in hand.

### BOOTS AND SHOES.

### Appointments.

ROTUNDA HOSPITAL.—Miss Hampson, who was trained at St. Thomas's Hospital, and held the post of Sister for three years in that hospital, has gone to the Rotunda Hospital, Dublin, as Matron.

CANTERBURY HOSPITAL.—Miss Messum, who was trained at St. Thomas's Hospital, and is Sister there, has been appointed Matron to the Canterbury Hospital.

VICTORIA INFIRMARY, LEWES.—Miss Ethel Lawrence, who trained at the Sussex County Hospital, has been appointed Matron at Lewes.

INDIAN SERVICE.—Miss Maud Mansell, late Superintendent Nurse of the Bishop Auckland District Nursing Association, has been appointed an Army Sister in the Indian Nursing Service.

BATFORD UNION HOSPITAL, NOTTINGHAM. — Miss S. Camilla Fletcher has been appointed Superintendent Nurse of the Batford Union Hospital, Nottingham. Miss Fletcher trained at York County Hospital and afterwards took charge of the Children's and Men's Surgical Wards at the Institution. She was then appointed Sister of the Women's and Children's Wards at Chichester Infirmary, and was afterwards working as Head Nurse at the Stockport Infirmary. She took her midwifery diploma, at Glasgow, December, 1889, and has since been engaged in private nursing in Nottingham and district where she gained wide popularity.

### Presentations.

THE officers and nurses of Toxteth Infirmary have presented Mr. T. M. Draper with an address and a salver and tea service, on the occasion of his resignation.

A VERY pleasant afternoon was spent at the Sarah Acland Home for Nurses, Oxford, on Monday afternoon, on the occasion of Mrs. Liddell leaving the town. She has been its President since its commencement in 1879. Tea was passed round by the nurses in their sitting-room, Sir Henry and Miss Acland being present. After tea Miss Denniston (Lady Superintendent) presented Mrs. Liddell with a handsome card-case, on which was engraved "To Mrs. Liddell, from the staff of the Sarah Ackland Nurses' Home, December, 1891." Mrs. Liddell thanked them in a very impressive manner, after which Sir Henry spoke to the nurses. The staff, which commenced with one nurse, now numbers 30, 5 of whom are engaged in nursing the sick poor in their own homes. The nurses' sitting-room was most tastefully decorated with choice flowers and plants, and music concluded the pleasant proceedings. Two of the nurses of this institution, Nurses Numcaster and Holbrook, have nursed the master of Balliol College (Dr. Jowett) who is now out of danger. The Dean and Mrs. Liddell leave for Ascot in January.

### Notes and Queries.

### Queries.

*Zinc Powder.*—What are the proper quantities of Oxide zinc and starch to make ordinary zinc and starch powder?

*Keating v. Dictionary.*—Can anyone tell a Nurse which would be the most useful for her to understand clearly the meaning of terms used by doctors, "Keating's Medical Lexicon," or "The Nurses' Dictionary," by Honnor Morten?

### Answers.

*Christmas Parcels.*—Received from Miss Lewis, Sister Goalen, Miss Pritchard Miss, M'Rae.

*Nursing in Prisons.*—There are infirmary wards for the male and female prisoners, and they are in charge of warders and wardresses. The dispenser of the prison seems to have chief control in the men's ward. On no account would trained nurses be permitted to enter them. The sick are very humanely treated.—*The Daughter of the Governor of a Prison.*

*Min.*—Male nurses are trained by the Hamilton Association, 57, Park Street, Grosvenor Square, W. Age 24.

*Lenah.*—The address in 27, Percy Street, Tottenham Court Road, W.C.

*Keating v. Dictionary.*—"The Nurses' Dictionary" is specially prepared for nurses' use, and so is best.

### The Message of the Chimes.

Over the silver mist that rose from meadow and vale, the moon was riding in the heavens. Some hours previously, the fiery, glowing sun had gone down, amid ruddy promises of a stern frost. So far, the youthful hopes which ran high of a real, old-fashioned Christmas pantomime of ice and snow were being realised. But the aged shivered and cowered somewhat, feebly wishing for a green Yule rather, even if it should render the kirkyard more populous. It is only in the veins of youth the blood can race fast enough to welcome gladly a carnival of skating and sliding, a Battle of Flowers, with snow for the blossoms.

In each and all of the homes that go to form the overgrown village of Shipley, the simmer of preparations had subsided into that hush before the storm which precedes great events. It was Christmas Eve, and, by-and-bye, the ringers would peal out the news of another holy birthday, from the ivied tower of the quaint, little Norman church that kept guard over the homes clustering close around it.

Under two roofs, however, there was another kind of stillness. Years ago, the two brothers Guest, Dr. John and Dr. James, were content to chum together. But, unhappily, these guardians of the village health had had a serious difference. The "bone of contention" had been that belonging to the arm which Farmer Joyce smashed when he came a cropper over an obstinate fence, out with the hounds. The brothers quarrelled fiercely over that arm, when erysipelas set in. Somehow, though the farmer was patched up, the quarrel of the brothers never had been. In fact, the "little rift" grew wider with the years, and threatened to "slowly silence all" between them.

Each of the doctors lived in an empty, silent home; the houses stood directly opposite to one another, in the narrow street, frowning stonily in that human way some houses have. The village had grown accustomed to the fact of the old medical firm being a divided one. Perhaps, so had the principals themselves. Though both of the doctors had a tolerable practice, Dr. John was immeasurably the richer man. Long years ago he had married, whereas Dr. James was a confirmed bachelor. True, both men were, to-day, equally alone and desolate, but Dr. John had priceless wealth stored up, in the shape of tender memories of that young mother and her tiny babe who had slipped away out of his life just when it was gladdest. Surely, it is better a thousand times to have loved and lost, as the poet sings, than never to have loved at all.

So, when Christmas Eve came round again, Dr. John was the happier of the two idle medical men—idle, because all ills must be staved off until the festive tide was passed and over—who sat each at a lonely fireside, hour after hour, until on the frosty, still air fell a clash and a peal.

"O peace and joy! O peace and joy!
Earth's only gold without alloy!"

The bells seemed to shout from out the ivied tower, and the echoes carried on the angelic melody.

Suddenly Dr. John rose and went to the window; in the house opposite the blinds were not shut, and somebody had, with a like instinct, been also drawn to gaze out. The lonely brothers faced each other.

"Poor old James!" murmured Dr. John. Then he walked back to the table, and stared down as a miser regards his gold, upon certain treasures lying in a small, open, metal casket. "Poor James!" the old man repeated, as he touched with tender, lingering fingers, two locks of hair which he periodically took out on certain anniversaries and on Christmas Eve.

We all have these secret and sacred hours for counting over our hidden gold, have we not? But what of those who have amassed no such wealth? It was late in the day for Dr. James to regret his poverty in that respect, but his straining eyes had a world of wistful longing in them as he looked across the belt of darkness into his brother's room, where the bending figure was accentuated against the brightness of lamp and fire light. What could John be looking at, he wondered? But when he saw the old man l ft something slowly to press his lips against it, Dr. James guess d; and his eyes grew even more wistfully hungry.

Over the way, Dr. John was murmuring:—

"Two locks—and they are wondrous fair—
Left me that vision mild:
The brown is from the mother's hair,
The blonde is from the child."

Their soft fragrance lingered about his lips, and as he laid gently down the brown tress and the soft little pinch of golden down, the chimes grew louder, and voices seemed to mingle with them. *Peace on earth!* Had that no meaning for the two lonely old men who lived lives rent asunder? Softly closing the casket, Dr. John impulsively crossed the street of darkness and the sullen gulf of estrangement in the same strides.

"A happy Christmas, James, old man!"

"The same to you, John!"

The trite greetings were over. The two old men stood, with clasped hands, their eyes finishing the reconciliation. Then they sat down by the fireside, and smoked the pipe of peace, with that apparent stolidity that distinguishes alike the cultured Briton and the noble savage.

A few hours later Shipley beheld an astonishing sight. Dr. John and Dr. James walked, arm in arm, to the little Norman church under the ivied tower, to sit, side by side, the first time for long years, in the old-fashioned Guest pew. For the estranged pair began, that Christmas morn, a new life—a life that will end, by and bye, for the two travellers, already so near the bottom of the hill, in that abode of perfect peace and waiting—Paradise.

### Amusements and Relaxation.

### SPECIAL NOTICE TO CORRESPONDENTS.

### Fourth Quarterly Word Competition commenced October 3rd, ends December 26th, 1891.

Competitors can enter for *all* quarterly competitions, but no competitor can take more than *one first prize* or *two* prizes of any kind during the year.

Proper names, abbreviations, foreign words, words of less than four letters, and repetitions are barred; plurals, and past and present participles of verbs, are allowed. Nuttall's *Standard* dictionary only to be used.

N.B.—Each paper must be signed by the author with his or her real name and address. A *nom de plume* may be added if the writer does not desire to be referred to by us by his real name. In the case of all prize-winners, however, the real name and address will be published.

The word for dissection for this, the THIRTEENTH week of the quarter, being

"MERRY XMAS."

| Names. | Dec. 19th. | Totals. | Names. | Dec. 19th. | Totals. |
|---|---|---|---|---|---|
| Lightowlers | 28 | 523 | Jenny Wren | 27 | 444 |
| Bonne | 27 | 523 | Darlington | 28 | 519 |
| Morico | 26 | 592 | Nurse G. P. | — | 99 |
| Robes | — | 143 | Hetty | 28 | 435 |
| Dulcamara | 27 | 536 | Janet | — | — |
| Psyche | — | 7 | Jackanapes | 28 | 367 |
| Agamemnon | 24 | 562 | Ex Nurse | — | 99 |
| Nurse J. S. | 25 | 499 | | | |

All letters referring to this page which do not arrive at 140, Strand, London, W.C. by the *first post on Thursdays*, and are not addressed PRIZE EDITOR, will in future be disqualified and disregarded.

## Figures and Tables

**Fig. 1. f1:**
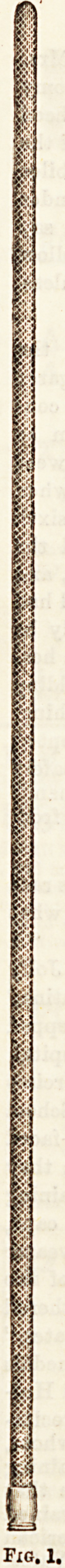


**Fig. 2. f2:**
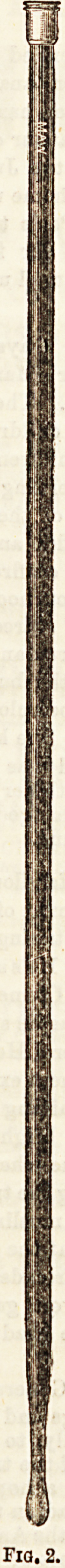


**Fig. 3. f3:**
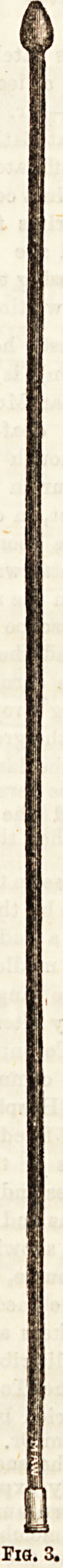


**Fig. 4. f4:**
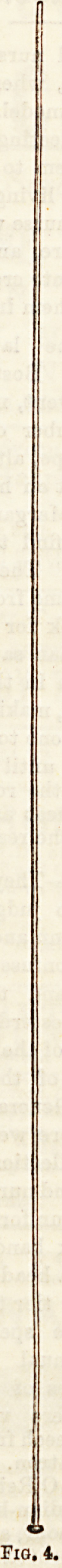


**Fig. 5. Fig. 6. Fig. 7. Fig. 8. f5:**
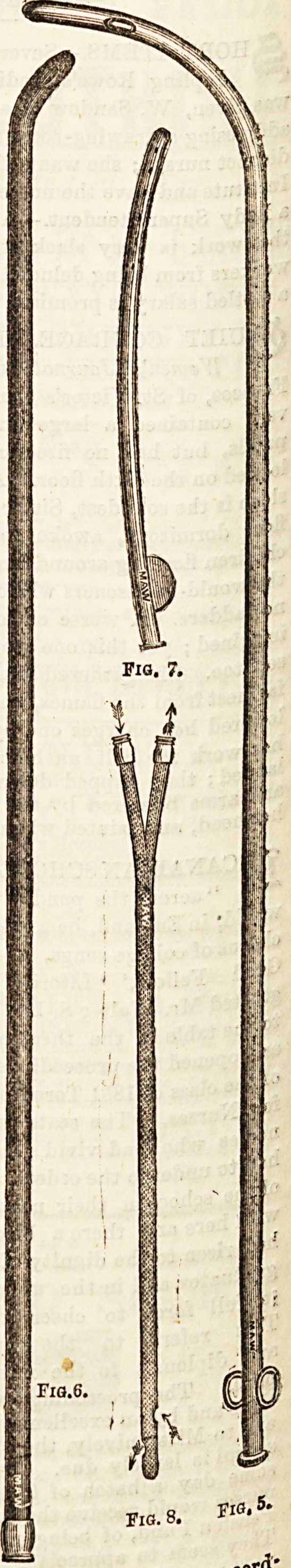


**Figure f6:**